# Atrio-ventricular coupling in patients with transposition of the great arteries after atrial switch by Magnetic Resonance Imaging

**DOI:** 10.1186/1532-429X-17-S1-Q94

**Published:** 2015-02-03

**Authors:** Magalie Ladouceur, Nadjia Kachenoura, Gilles Soulat, Emilie Bollache, Alban Redheuil, Christophe Delclaux, Gilles Chatellier, Michel Azizi, Pierre Boutouyrie, Damien Bonnet, Laurence Iserin, Elie Mousseaux

**Affiliations:** 1 Cardiology, Hôpital Européen Georges Pompidou, Paris, France; 2 Inserm U970, Paris, France; 3 Inserm, Paris, France; 4 Radiology, Hôpital Européen Georges Pompidou, Paris, France; 5 Institut de Cardiologie, Pitié-Salpétrière, Paris, France; 6 Hôpital Européen Georges Pompidou, Paris, France; 7 Necker, Paris, France

## Background

Diastolic function has been little explored in d-transposition of the great arteries palliated by atrial switch (d-TGA). However, early detection of diastolic dysfunction could be of major clinical interest and could explain clinical status in this population. We aimed: 1/ to characterize diastolic function in d-TGA after atrial switch from velocity encoded cardiac magnetic resonance (CMR) data and 2/ to study associations between diastolic function indices and objective measurements of clinical status evaluated by exercise test.

## Methods

Forty five patients (11 females; mean age 32±4 years) with atrially switched d-TGA and 45 healthy subjects matched for age and sex were prospectively included. CMR examination was performed in both groups, and cardio-pulmonary exercise test was performed within 24 hours in patients. CMR tricuspid, mitral inflow and systemic ventricular outflow tract velocities and flow-rates were analyzed using custom software to estimate diastolic parameters (figure). Pulmonary vein baffle, atrial areas and right atrial strain as [(maximal atrial area - minimal atrial area) / maximal atrial area] were also measured.

**Figure 1:**
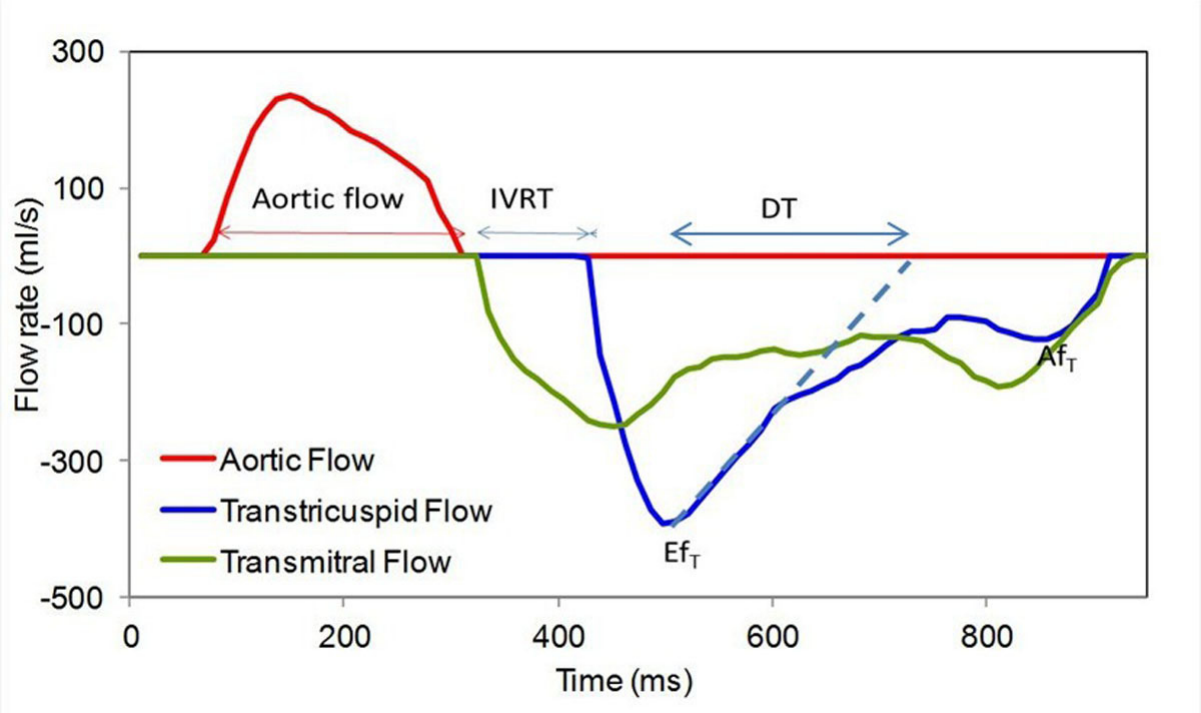


## Results

CMR diastolic parameters were significantly different in d-TGA patients as compared to controls. In d-TGA patients, parameters of systemic right ventricular (RV) relaxation were markedly impaired compared to left ventricle of controls, as reflected by a significant increase in deceleration and isovolumic relaxation times (p<0.01). Early trans-tricuspid peak filling-rate (Ef) and RV filling duration were negatively correlated with systemic RV remodeling index (Mass/volume) (r=-0.38 and -0.45, respectively, p<0.01). Systemic RV stroke volume and Ef were significantly associated with baffle size (r=0.35 and r=0.33, p≤0.02, respectively). While peak VO2 was not related to systemic RV volumes, RVEF and to RV Mass, it was significantly associated with systemic ventricle filling indices, such as Ef/filling volume, A wave, filling volume. In multivariate analysis, a significant association was found between RA strain and exercise peak oxygen uptake (r=0.42, p<0.01) in d-TGA patients, while systemic RV ejection fraction was not correlated with exercise performances. Such associations were independent of age, gender and body mass index.

## Conclusions

In d-TGA after atrial switch, systemic RV exposed to systolic dysfunction can be also characterized by an early impairment of diastolic function. Systemic RV diastolic dysfunction is characterized by abnormal RV relaxation that limits cardiac output augmentation.

## Funding

This work was supported by Assistance Publique Hôpitaux de Paris. The authors were totally independent of the funder for all scientific aspects of the research.

